# Unusual Presentation of a Rare Tumor: Histiocytic Sarcoma Presenting as a Finger Growth

**DOI:** 10.7759/cureus.6150

**Published:** 2019-11-13

**Authors:** Zafar Ali, Faiza Hanif

**Affiliations:** 1 Histopathology, Shifa International Hospital, Islamabad, PAK; 2 Pathology, Shifa International Hospital, Islamabad, PAK

**Keywords:** finger, histiocytic sarcoma, bone

## Abstract

Histiocytic sarcoma (HS) is a malignant neoplasm with histiocytic differentiation. It presents most commonly at extranodal sites. We report a case of HS in a 15-year-old female with a history of trauma to the right little finger. Radiograph of the hand was reported as malignant tumor primarily arising from bone and invading soft tissues. Histologically, tumor cells are characterized by abundant eosinophilic cytoplasm and eccentric round to oval nuclei with atypia. One or more small and distinct nucleoli are present. The tumor cells were positive for CD68, S100, CD4 and lymphocyte common antigen, while epithelial membrane antigen, HMB-45, CK AE1/AE3, myogenin, desmin, CD1a, CD21 and SALL-4 were negative thus ruling out rhabdomyosarcoma, extrarenal rhabdoid tumor, Langerhans cell sarcoma and malignant melanoma.

## Introduction

Histiocytic sarcoma (HS) is derived from cells of the monocyte/macrophage lineage. It is a rare tumor of mature histiocytes and can arise from low-grade B-cell lymphomas in rare cases. Most of the patients are adults with a median age of 46 years [1]. Its clinical presentation and morphological features mimic other lymphoid tissue malignancies, i.e. B symptoms (fever, night sweats, 10% or more weight loss), lymphadenopathy hepatosplenomegaly and peripheral blood cytopenia [2]. It has also been reported as a second malignancy after chemotherapy given for germ cell tumors [3]. The tumor cells are large, non-cohesive with abundant eosinophilic cytoplasm. The nuclei contain one or more distinct nucleoli and vesicular chromatin. The most common sites of presentation are extranodal sites including gastrointestinal tract, skin and soft tissues. HS is an aggressive tumor with 50% mortality. The standard treatment is surgery. Adjuvant radiotherapy and chemotherapy are also used [4]. Here we present an unusual case of HS presenting as a finger growth.

## Case presentation

**Figure 1:**
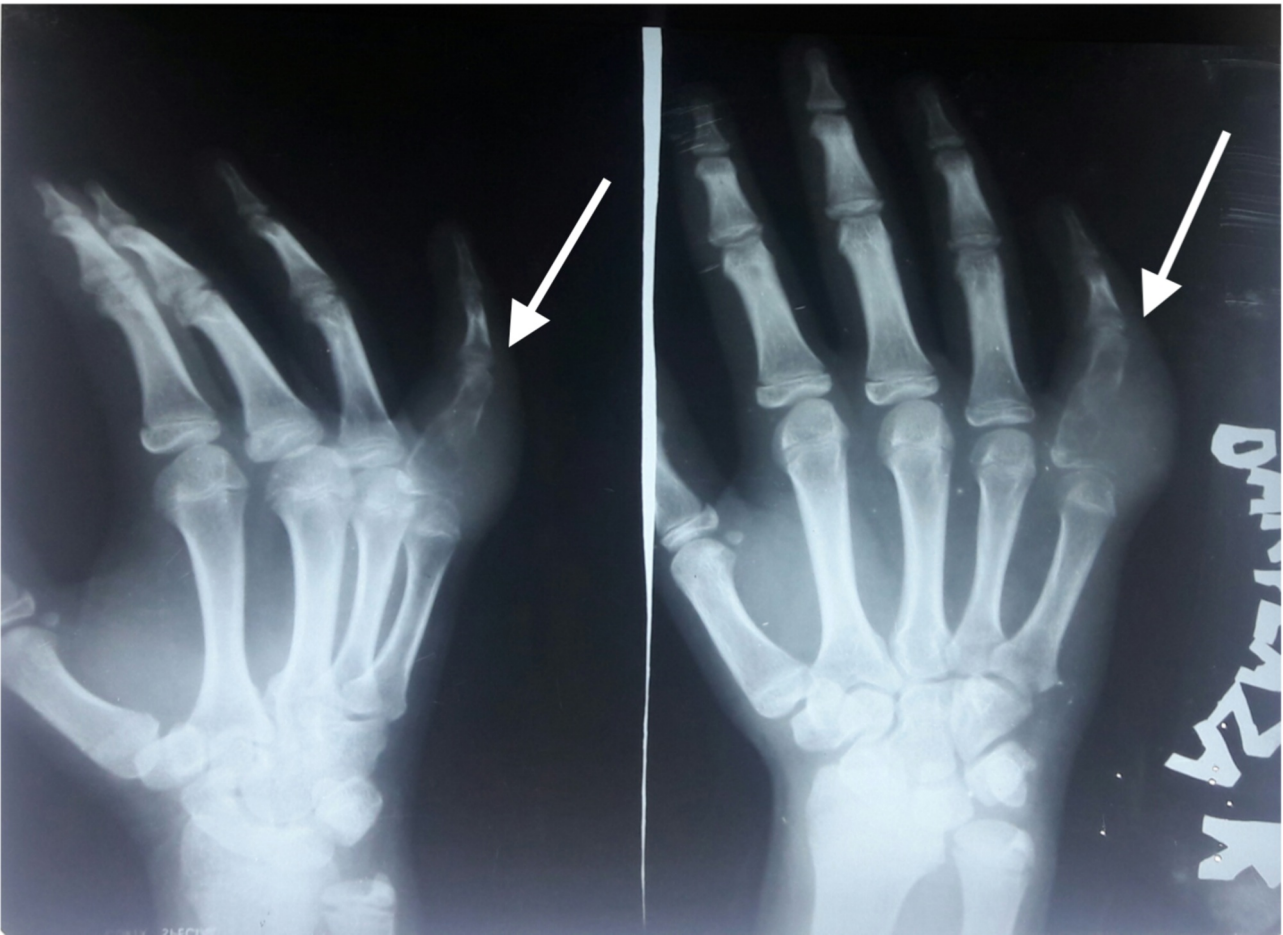
X-rays showing a malignant tumor originating from bone and infiltrating the soft tissue (white arrow).

A 15-year-old female child presented with history of trauma to the right little finger. Radiograph of the right hand showed a malignant tumor originating from the bone and infiltrating the soft tissues (Figure 1).

**Figure 2:**
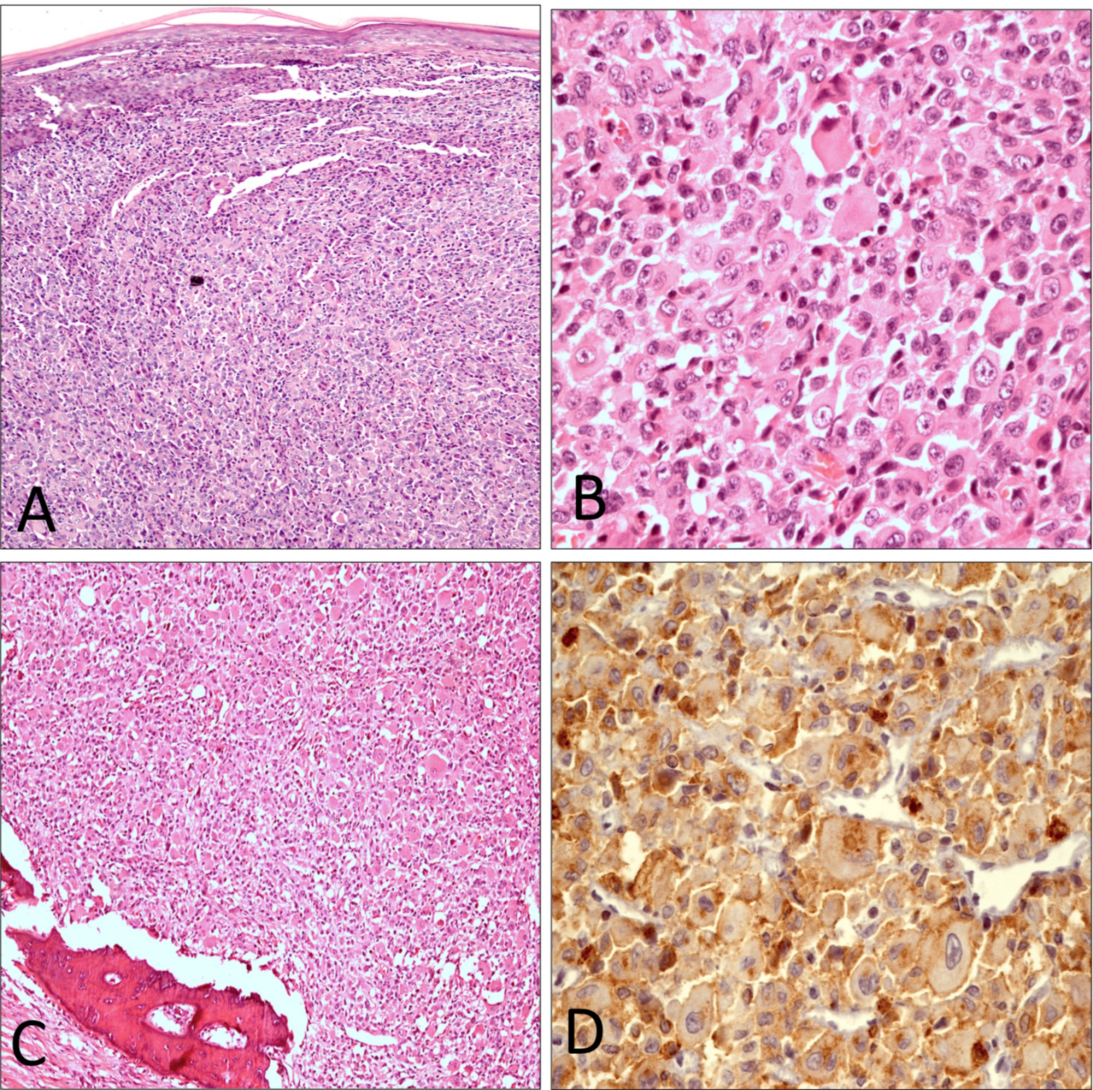
(A) Low-power view showing sheets of cells with plump eosinophilic cytoplasm (hematoxylin and eosin x40). (B) High-power view showing atypical histiocytes having prominent nucleoli and admixed inflammatory cells (hematoxylin and eosin x400). (C) Tumor cells infiltrating bony tissue (hematoxylin and eosin x100). (D) Strong positive CD68 immunostaining (x400).

Amputation of the right little finger upto mid metacarpal was performed. On gross examination, the overlying skin was intact with no involvement by the tumor. The tumor measured 6.2 x 3.0 x 2.2 cm. Cut surface of the tumor shows homogeneous, tan white appearance with bone involvement. The resection margin was grossly uninvolved. The histological findings reveal diffuse non-cohesive proliferation of large round to oval cells. The cells have vesicular, round to oval nuclei with moderate atypia and abundant eosinophilic cytoplasm. At these areas, spindle cell differentiation is seen. Admixed small lymphocytes, plasma cells and eosinophils are identified. The tumor is invading the bone. The sections were stained with a panel of monoclonal antibodies. The tumor cells are positive for CD68, S100, CD4 and lymphocyte common antigen, while negative for epithelial membrane antigen, HMB-45, CK AE1/AE3, myogenin, desmin, CD1a, CD21 and SALL-4 (Figure 2A-2D).

On the basis of morphological features and immunohistochemical findings, a diagnosis of HS was rendered.

## Discussion

HS is an aggressive neoplasm with a poor response to therapy. Patient’s prognosis depends on the extent of disease and size of the tumor. Clinical presentation varies depending on the site of involvement. Most commonly involved organs are intestine, skin, spleen, lymph nodes and bone marrow [5,6]. In our case, the patient presented with the growth of the right little finger with a history of trauma to the digit. This is a very rare presentation of HS.

Mathé et al. first described the histological features of HS [7]. These features remain important but currently greater emphasis is placed on immunohistochemical and genetic features for diagnosis. HS characteristically expresses one or more histiocytic markers. They do not express B-cell, T-cell, or myeloid markers. The diagnosis is based on specific histiocytic origin immunostains, i.e. CD68, lysozyme, α1-antitrypsin and CD163 [8]. Considering the age of the patient, differentials included rhabdomyosarcoma, extrarenal rhabdoid tumor, Langerhans cell sarcoma, epithelioid sarcoma, HS and melanoma. So a panel of immunohistochemical markers was used to reach to the diagnosis.

The preferred treatment for localized disease is surgical resection and adjuvant radiation, while for aggressive or multifocal presentations it is a combination of chemotherapy and bone marrow transplant [9,10]. In our case, the tumor is >3.5 cm, localized and completely resected. The patient did not receive any treatment. A follow-up showed no recurrence, and the patient is doing well.

## Conclusions

HS is a challenging diagnosis, as it has a broad morphological differential diagnosis. Conclusive diagnosis requires attention to morphological details and judicious use of immunohistochemical stains. It usually pursues an aggressive clinical course; however, solitary localized lesions with early complete excision may have favorable outcomes. A combination of lymphoid and histiocytic lineage immunostains helps in reaching to a final diagnosis.
